# Simultaneous stimulation of glycolysis and gluconeogenesis by feeding in the anterior intestine of the omnivorous GIFT tilapia, *Oreochromis niloticus*

**DOI:** 10.1242/bio.024836

**Published:** 2017-06-15

**Authors:** Yong-Jun Chen, Ti-Yin Zhang, Hai-Yan Chen, Shi-Mei Lin, Li Luo, De-Shou Wang

**Affiliations:** 1 Key Laboratory of Freshwater Fish Resources and Reproductive Development (Ministry of Education), College of Animal Science and Technology, Southwest University, Chongqing 400715, China; 2 Key Laboratory of Aquatic Science of Chongqing, School of Life Sciences, Southwest University, Chongqing 400715, China

**Keywords:** Anterior intestine, Glucose metabolism, Role, Feeding, Tilapia

## Abstract

The present study was performed to investigate the roles of anterior intestine in the postprandial glucose homeostasis of the omnivorous Genetically Improved Farmed Tilapia (GIFT). Sub-adult fish (about 173 g) were sampled at 0, 1, 3, 8 and 24 h post feeding (HPF) after 36 h of food deprivation, and the time course of changes in intestinal glucose transport, glycolysis, glycogenesis and gluconeogenesis at the transcription and enzyme activity level, as well as plasma glucose contents, were analyzed. Compared with 0 HPF (fasting for 36 h), the mRNA levels of both ATP-dependent sodium/glucose cotransporter 1 and facilitated glucose transporter 2 increased during 1-3 HPF, decreased at 8 HPF and then leveled off. These results indicated that intestinal uptake of glucose and its transport across the intestine to blood mainly occurred during 1-3 HPF, which subsequently resulted in the increase of plasma glucose level at the same time. Intestinal glycolysis was stimulated during 1-3 HPF, while glucose storage as glycogen was induced during 3-8 HPF. Unexpectedly, intestinal gluconeogenesis (IGNG) was also strongly induced during 1-3 HPF at the state of nutrient assimilation. The mRNA abundance and enzyme activities of glutamic-pyruvic and glutamic-oxaloacetic transaminases increased during 1-3 HPF, suggesting that the precursors of IGNG might originate from some amino acids. Taken together, it was concluded that the anterior intestine played an important role in the regulation of postprandial glucose homeostasis in omnivorous tilapia, as it represented significant glycolytic potential and glucose storage. It was interesting that postprandial IGNG was stimulated by feeding temporarily, and its biological significance remains to be elucidated in fish.

## Abstract (precis)

**Summary:** Simultaneous stimulation of glycolysis and gluconeogenesis by feeding in the anterior intestine of the omnivorous GIFT tilapia, *Oreochromis niloticus*.

## INTRODUCTION

Besides liver and kidney, small intestine is identified as the third gluconeogenic organ to contribute to endogenous glucose production (EGP) in the post-absorptive or fasting state of mammals (Croset et al., 2001; Mithieux et al., 2004a). Intestinal gluconeogenesis (IGNG) might account for about 5–7% of EGP in the post-absorptive state in rats (*Rattus norvegicus*) fed with a regular chow diet (Mithieux et al., 2006). In mammals, small intestine is known to be an insulin-sensitive organ (Mithieux, 2009), and portal sensing of IGNG is a mechanistic link in the diminution of food intake induced by a protein-enriched diet (Mithieux et al., 2005). However, IGNG is not modified with low dietary protein reception in the carnivorous rainbow trout (*Oncorhynchus mykiss*) (Kirchner et al., 2005). In addition, both *in vivo* and *in vitro* data prove that a regulation of IGNG by insulin, glucose, lactate or amino acid changes is absent in rainbow trout (Polakof et al., 2010). It seems that the role of IGNG in glucose homeostasis and its regulatory mechanisms between fish and mammals might be very different.

Similar with mammals, the intestine is evidenced with significant glycolytic potential in fish (Anderson, 1974; Polakof et al., 2010). Glucose uptake across the fish intestine is much higher than in any other tissue except the brain (Blasco et al., 1996, 2001), and hyperglycemia induced by a glucose load provokes an increase in glucose uptake across the intestine (Blasco et al., 2001). Glucose uptake in fish intestine is mediated by a sodium/glucose cotransporter (Sglt, also called as Slc5a) present in the brush border membrane of enterocytes, which is followed by a facilitated diffusion through glucose transporter 2 (Glut2, also called as Slc2a2) across the basolateral membrane to the blood (Drai et al., 1990; Reshkin and Ahearn, 1987). In carnivorous rainbow trout, the mRNA level of intestinal *sglt1* rather than *glut2* increased in response to a glucose load (Kirchner et al., 2008; Polakof et al., 2010). In addition, enhanced glucose phosphorylation, glycogen storage as well as glucose oxidation of mid-gut were associated with a glucose load (Polakof et al., 2010; Soengas et al., 2006). Existing literature have generally proved that gut plays an important role in the glucose homeostasis of carnivorous rainbow trout, while information in omnivorous or herbivorous fish is very limited. Considering that the glucose clearance rate of carnivorous fish receiving a carbohydrate-enriched diet is much lower than that of omnivorous or herbivorous fish (Moon, 2001; Hemre et al., 2002), we hypothesize that the gut might represent significant potentials of glucose utilization in the omnivorous or herbivorous fish, and the response time of the gut to utilize dietary glucose might be much faster as compared with carnivorous fish. Thus, it is necessary to perform a time course study of intestinal glucose metabolism in omnivorous or herbivorous fish after a meal.

As a typical omnivorous fish, tilapia is the second most farmed fish group worldwide due to its rapid growth, strong disease resistance, high marketability and relatively stable market price (Ng and Romano, 2013). In 2014, the total yield of Nile tilapia in the world was about 3.67 million metric tons, and China accounted for 34.8% of the production (http://www.fao.org/fishery/statistics/global-aquaculture-production/query/en, accessed on 5th March 2017). The Genetically Improved Farmed Tilapia (GIFT) strain of *O. niloticus* was developed by the International Center for Living Aquatic Resources Management (ICLARM) from selected breeding stocks from Africa and Asia (Tendencia et al., 2006), and it has been widely cultured in China in recent years. The genome sequence of tilapia is already available (Brawand et al., 2014), which makes it a good candidate to study the regulatory mechanisms of nutrients for omnivorous fish species. Thus, this study was performed to investigate the time course of changes in the intestinal glucose transport and utilization at the transcription and enzymatic activity level of tilapia after a meal. The results of this study would help us to ascertain the role of anterior intestine in the postprandial glucose homeostasis of omnivorous fish.

## RESULTS

### Glucose transport and plasma glucose level

**Fig. 1.**
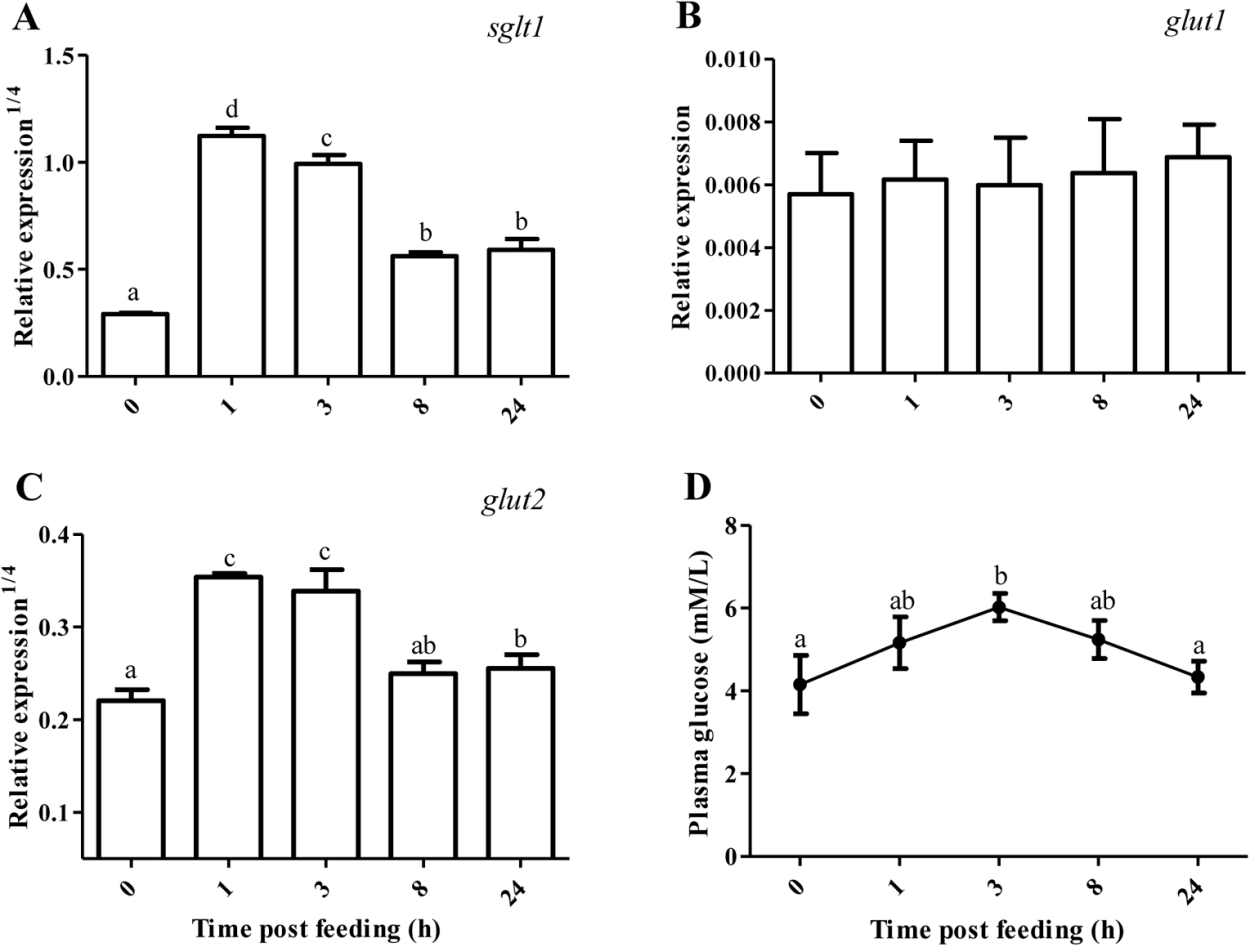
**Relative expression of intestinal glucose transporters and plasma glucose level of tilapia.** Values represent means±s.d. of six replicates (*n*=6), and values with different letters above the error bar indicate significant differences between treatments (*P*<0.05; Tukey's test). (A) *sglt1*, sodium/glucose cotransporter 1; (B) *glut1*, glucose transporter 1; (C) *glut2*; (D) glucose.

The mRNA level of intestinal glucose transporter and plasma glucose level in tilapia are shown in Fig. 1. After a regular PCR amplification, *glut4* was found to be scarcely expressed in the anterior intestine based on gel electrophoresis analysis. Compared with 0 h post feeding (HPF) (fasting for 36 h), the mRNA level of *sglt1* sharply increased during 1-3 HPF (*P*<0.05), decreased at 8 HPF (*P*<0.05) and then leveled off. The expression of *glut1* (0.0062, average value of five different sampling time points) was comparable to that of *glut2* (0.0074). Although the mRNA level of *glut1* was not changed with feeding time (*P*>0.05), *glut2* mRNA abundance increased by 4.61-5.54 times during 1-3 HPF (*P*<0.05), and then decreased to basal level at 8-24 HPF as compared with 0 HPF. Compared with basal level of 4.15 mM/l at 0 HPF, plasma glucose level trended to increase at 1 HPF (5.16 mM/l), reached maximal value of 6.02 mM/l at 3 HPF (*P*<0.05), decreased at 8 HPF, and then returned to basal level at 24 HPF.

### Glucose metabolism

**Fig. 2.**
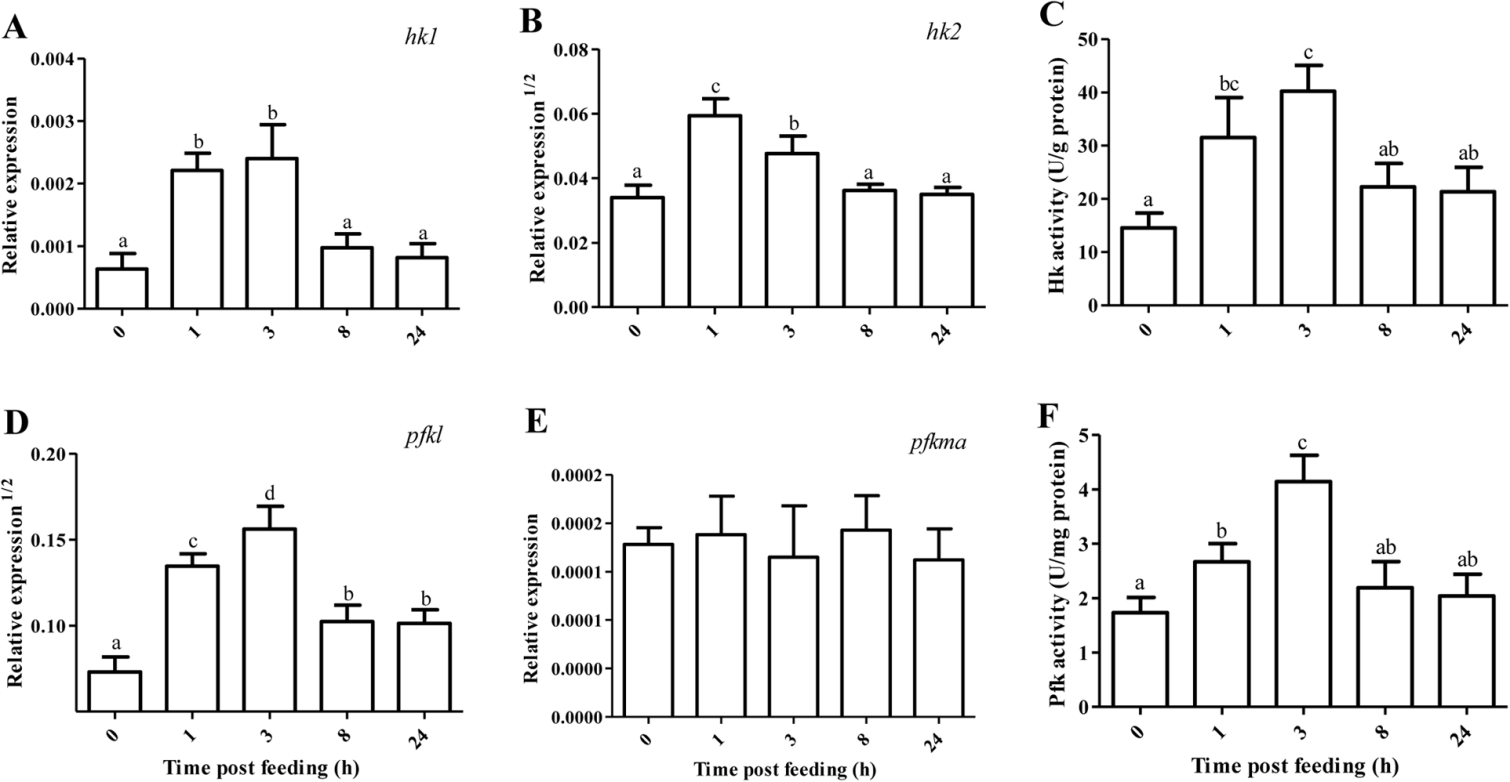
**Relative expression of glycolytic gene and its enzymatic activity in the anterior intestine of tilapia.** Values represent means±s.d. of six replicates (*n*=6), and values with different letters above the error bar indicate significant differences between treatments (*P*<0.05; Tukey's test). (A) *hk1*, hexokinase 1; (B) *hk2*; (C) Hk; (D) *pfkl*, phosphofructokinase (liver type); (E) *pfkma*, phosphofructokinase (muscle type a); (F) Pfk.

The mRNA level of intestinal key glycolytic gene and its enzymatic activity are presented in Fig. 2. After a regular PCR amplification, *gck* and *pfkmb* were found to be scarcely expressed in the anterior intestine of tilapia. The expression of *hk1* (0.0013) was a little lower than that of *hk2* (0.0018). The mRNA level of *hk1* was not differentiated between 1 and 3 HPF (*P*>0.05), but it was significantly higher than that of the other feeding time (*P*<0.05). Compared with 0 HPF, *hk2* mRNA abundance increased by 0.97-2.03 times during 1-3 HPF (*P*<0.05), and was then reversed after 8 h of feeding. The activity of Hk significantly increased at 1 HPF (*P*<0.05), reached maximum at 3 HPF, and then returned to basal level when the feeding time was over 8 h. The expression of *pfkl* (0.013) was about 72.2 times higher than that of *pfkma* (0.00018). The mRNA level of *pfkl* significantly increased by 2.37-3.54 times during 1-3 HPF (*P*<0.05), markedly decreased at 8 HPF and then leveled off. However, *pfkma* mRNA abundance was not changed with feeding time (*P*>0.05). The activity of Pfk increased by 54-139% during 1-3 HPF (*P*<0.05), and then was reversed after 8 h of feeding.

**Fig. 3.**
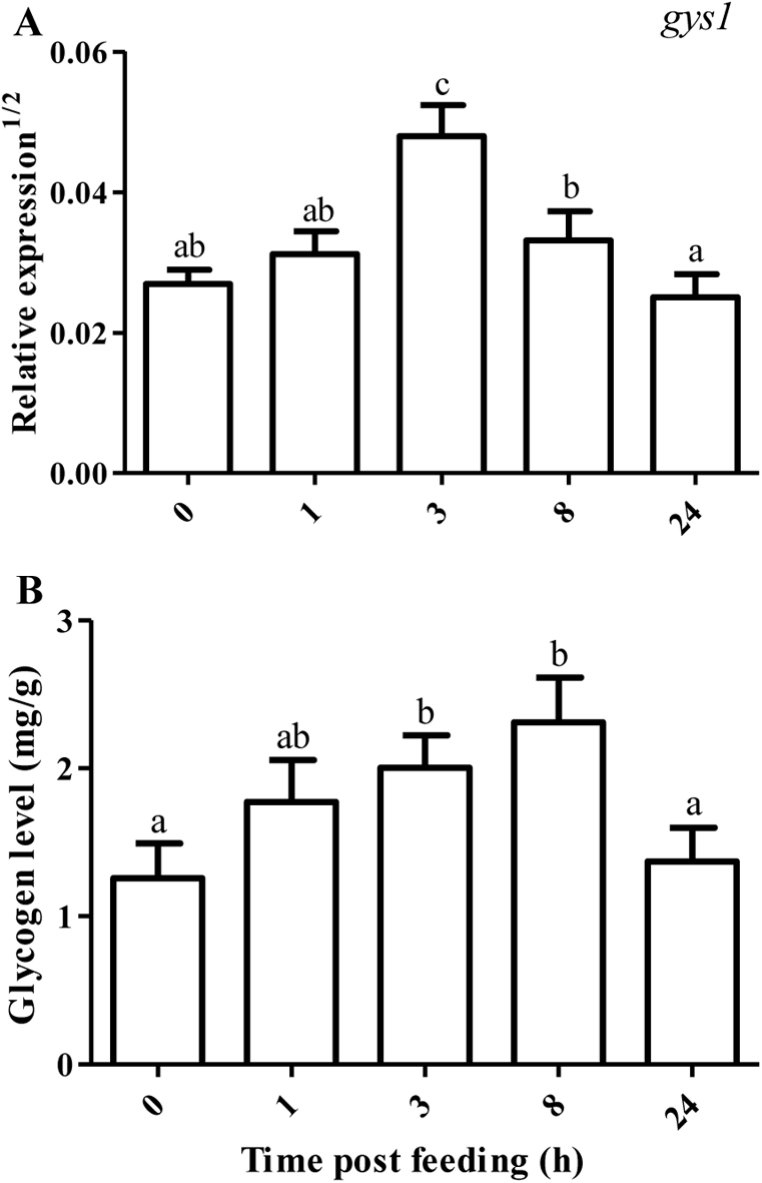
**Relative expression of glycogen synthase 1 and glycogen level in the anterior intestine of tilapia.** Values represent means±s.d. of six replicates (*n*=6), and values with different letters above the error bar indicate significant differences between treatments (*P*<0.05; Tukey's test). (A) *gys1*, glycogen synthase 1; (B) glycogen.

After a regular PCR amplification, *gys2* was found to be scarcely expressed in the anterior intestine of tilapia. As detailed in Fig. 3, the mRNA level of *gys1* did not significantly increase until the feeding time was over 3 h (*P*<0.05), then it was reversed after 8 h of feeding as compared with 0 HPF (*P*<0.05). The glycogen content trended to increase at 1 HPF, significantly increased at 3-8 HPF (*P*<0.05), and returned to basal level at 24 HPF.

**Fig. 4.**
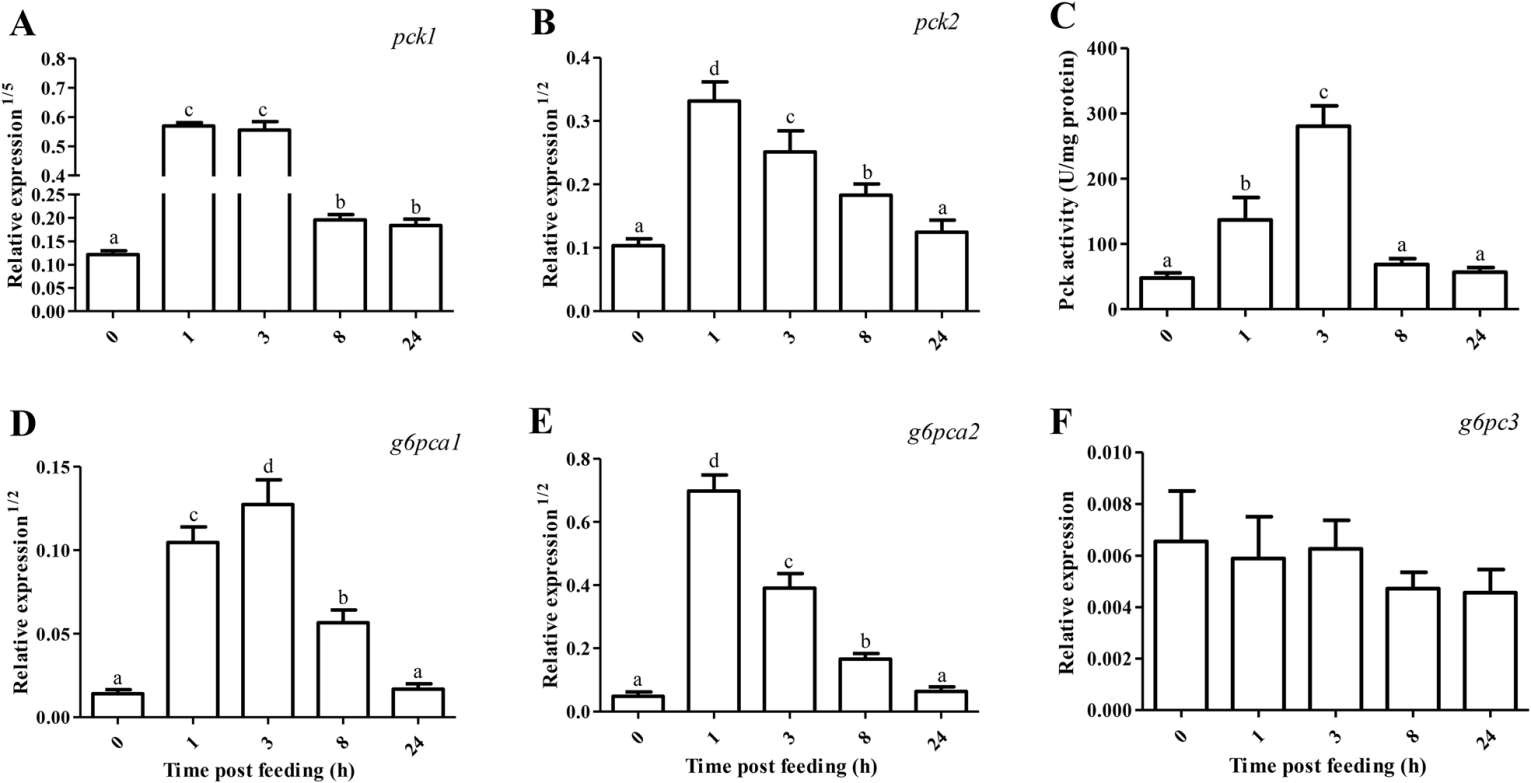
**Relative expression of gluconeogenic gene and its enzymatic activity in the anterior intestine of tilapia.** Values represent means±s.d. of six replicates (*n*=6), and values with different letters above the error bar indicate significant differences between treatments (*P*<0.05; Tukey's test). (A) *pck1*, phosphoenolpyruvate carboxykinase 1; (B) *pck2*; (C) Pck; (D) *g6pca1*, glucose-6-phosphatase catalytic subunit a1; (E) *g6pca2*; (F) *g6pc3*.

The expression of gluconeogenic gene and its enzymatic activity in the anterior intestine of tilapia are shown in Fig. 4. After a regular PCR amplification, *g6pc2* was found to be scarcely expressed in the intestine. The expression of *pck1* (0.019) was about 1.15 times lower than that of *pck2* (0.041). The highest mRNA level of *pck1* was observed at 1 and 3 HPF, followed by 8 and 24 HPF, and the lowest at 0 HPF (*P*<0.05). Compared with 0 HPF, *pck2* mRNA abundance increased by 4.93-9.30 times during 1-3 HPF (*P*<0.05), decreased at 8 HPF (*P*<0.05), and then returned to basal level at 24 HPF. The activity of Pck was markedly stimulated at 1 HPF (*P*<0.05), reached maximum at 3 HPF, and then was reversed after 8 h of feeding. The expression of *g6pca1* (0.0052) was about 20.7 times lower than that of *g6pca2* (0.11). The mRNA level of *g6pca1* markedly increased by 52.8-79.0 times during 1-3 HPF (*P*<0.05), decreased at 8 HPF (*P*<0.05), and then returned to basal level at 24 HPF. The expression of *g6pca2* was regulated at a similar pattern as that of *pck2*. The mRNA abundance of *g6pc3* was not impacted by feeding time (*P*>0.05).

### Amino acid metabolism

**Fig. 5.**
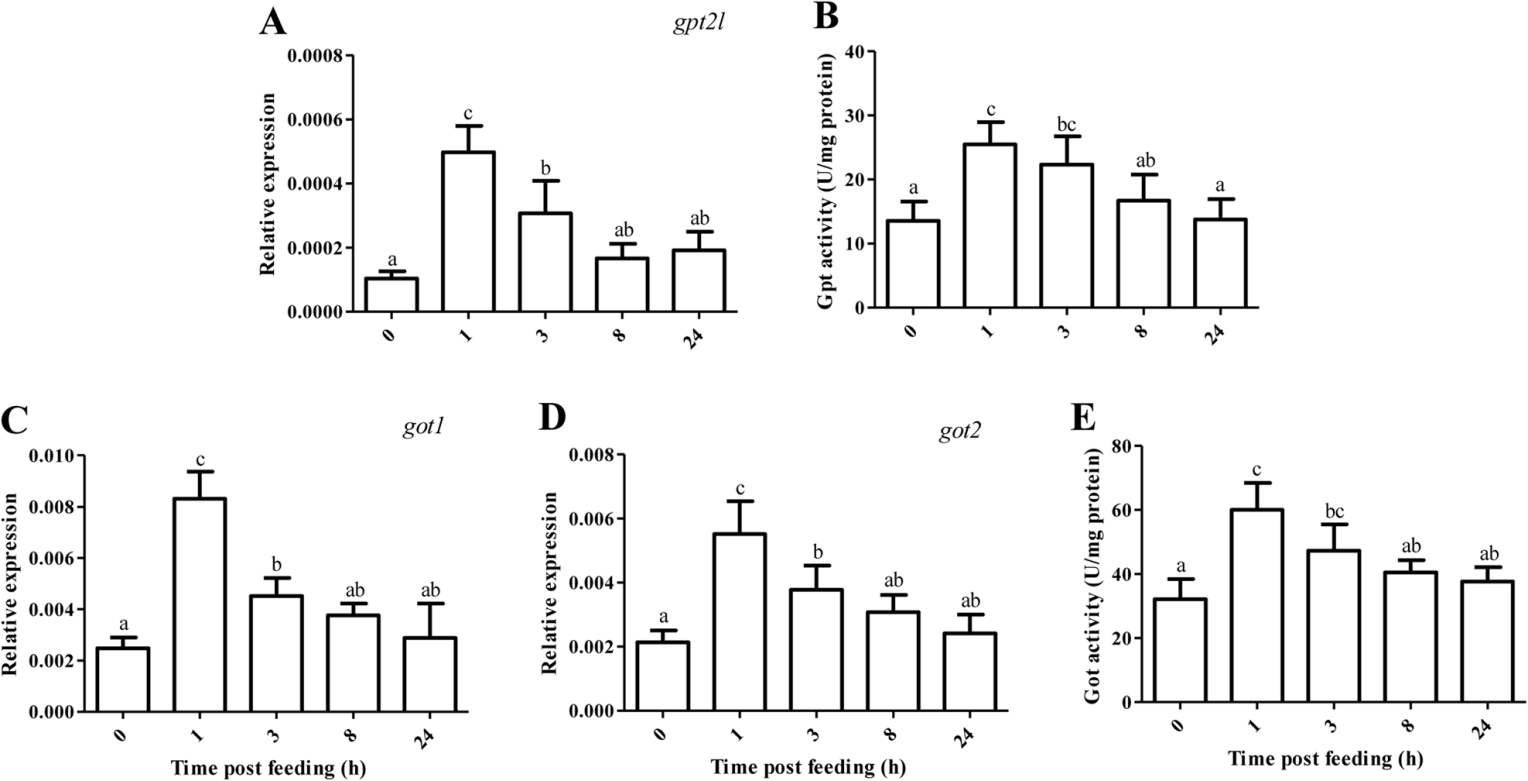
**Relative expression of transaminase and its enzymatic activity in the anterior intestine of tilapia.** Values represent means±s.d. of six replicates (*n*=6), and values with different letters above the error bar indicate significant differences between treatments (*P*<0.05; Tukey's test). (A) *gpt2l*, glutamic-pyruvic transaminase 2-like; (B) Gpt; (C) *got1*, glutamic-oxaloacetic transaminase 1; (D) *got2*; (E) Got.

The mRNA level of aminotransferase and its enzymatic activity in the anterior intestine of tilapia are shown in Fig. 5. After a regular PCR amplification, *gpt1* was found to be scarcely expressed in the intestine. The mRNA level of *gpt2l* (glutamic-pyruvic transaminase 2-like) increased by 1.98-3.84 times during 1-3 HPF (*P*<0.05), and it was reversed after 8 h of feeding as compared with 0 HPF. Correspondingly, the activity of Gpt increased by 65-88% during 1-3 HPF compared with that of 0 HPF (*P*<0.05). The regulation of *got1* and *got2* expression by feeding time generally followed similar pattern as that of *gpt2l*, and a temporary increase of Got activity was also recorded during 1-3 HPF.

## DISCUSSION

To the best of our knowledge, this is the first report to systematically evaluate the glucose metabolism of intestine at the molecular level in an omnivorous fish species. In this study, the mRNA level of *glut1* was not modified by feeding time, suggesting that *glut1* might be responsible for the low level of basal glucose uptake required to sustain respiration in the enterocytes of tilapia as reported in mammals (Zhao and Keating, 2007). The expression of ATP-dependent *sglt1* sharply increased by 134-220 times while the mRNA level of facilitated *glut2* increased by 4.61-5.54 times during 1-3 HPF in the anterior intestine of tilapia as compared with 0 HPF (fasting for 36 h). However, the mRNA abundance of both *sglt1* and *glut2* decreased with feeding time from 3 to 8 h, and then leveled off. These results indicated that intestinal uptake of glucose and its transport across the basolateral membrane of enterocytes to the blood mainly occurred during 1-3 HPF, which subsequently contributed to the increase of plasma glucose level at the same time. Thus, postprandial nutritional status transition from nutrient assimilation to post-absorptive period in the intestine occurred at about 8 HPF. In line with this study, a glucose load was presumably to activate glucose transport rates across the intestine through both Sglt and Glut2 in omnivorous black bullhead (Soengas and Moon, 1998). However, the mRNA level of intestinal *sglt1* rather than *glut2* increased in response to a glucose load in carnivorous rainbow trout (Kirchner et al., 2008; Polakof et al., 2010).

HK and PFK act as two key regulatory enzymes of the glycolytic pathway (Robey and Hay, 2006; Wegener and Krause, 2002). In the present study, increased mRNA abundance of intestinal *hk1* (by 2.48-2.80 times), *hk2* (by 0.97-2.04 times) and *pfkl* (by 2.37-3.54 times) were recorded during 1-3 HPF, and the expression of all these glycolytic genes decreased when the nutritional condition transited to the post-absorptive period at 8 HPF. In addition, the activities of Hk and Pfkl paralleled with the mRNA levels of their corresponding encoding genes, suggesting that glycolysis was activated during 1-3 HPF in the anterior intestine of omnivorous tilapia. In accordance with this study, intestine tissue was also evidenced with significant glycolytic potential in omnivorous black bullhead (Soengas and Moon, 1998) and carnivorous trout (Blasco et al., 2001; Polakof et al., 2010) as well as in mammals (Anderson, 1974). In the present study, *pfkmb* was scarcely expressed in the intestine of tilapia. The mRNA abundance of *pfkl* was superior to that of *pfkma*, and the expression of *pfkma* was not changed with postprandial feeding time. It was postulated that *pfkl* might be the most important and functional *pfk* paralogs in the regulation of intestinal glycolysis of tilapia.

In this study, *gys2* was scarcely expressed in the intestine of tilapia. However, the mRNA abundance of *gys2* dominated over that of *gys1* in the liver (Chen et al., 2017). It seemed that there was a different tissue distribution between *gys1* and *gys2*. Intestinal *gys1* mRNA level increased by 2.41 times at 3 HPF, and then decreased at 8 HPF. Intestinal glycogen level also increased at 3 HPF, but it maintained stable at 8 HPF. These results suggesting anterior intestine had the capacity to store glucose as glycogen, and glycogen accumulated during 3-8 h after a meal in omnivorous tilapia. In carnivorous rainbow trout, intestinal glycogen content also increased in response to a glucose load, and it was mostly located in the muscle layers of intestine (Polakof et al., 2010; Soengas et al., 2006).

PCK is a key enzyme in the regulation of gluconeogenesis (Nordlie et al., 1999), and it is only regulated at the transcription level for its enzyme production (Hanson and Reshef, 1997). In the present study, the mRNA levels of intestinal cytosolic *pck1* (by 1983-2227 times) and mitochondrial *pck2* (by 4.93-9.30 times), as well as Pck activity, were all stimulated during1-3 HPF, but they decreased at 8 HPF when the nutritional condition transited to the post-absorptive period. These results were contrary to those of liver, in which *pck2* mRNA level sharply decreased during 1-3 HPF, but it rose with feeding time from 3 to 8 h (Chen et al., 2017). The results regarding postprandial changes in *pck* mRNA level and its activity in the intestine of tilapia also differed from data obtained in rats and carnivorous fish. In rats, both the expression of cytosolic PCK1 and its activity were stimulated at the post-absorptive state (Rajas et al., 2000). The activity of Pck rather than its mRNA abundance increased in the intestine of food-deprived rainbow trout for 14 days (Kirchner et al., 2008).

Glucose-6-phosphatase (G6Pase) catalyzes the hydrolysis of glucose-6 phosphate to glucose and inorganic phosphate, which is the last biochemical reaction common to both gluconeogenesis and glycogenolysis (Hutton and O'Brien, 2009; van de Werve et al., 2000). Regulation of G6PC mRNA abundance is a major control of G6Pase activity (Argaud et al., 1996). In this study, the mRNA levels of both intestinal *g6pca1* (by 52.8-79.0 times) and *g6pca2* (by 62-198 times) sharply increased during 1-3 HPF, but they decreased at 8 HPF when the nutritional condition transited to the post-absorptive period. In rats, however, both the expression of *G6PC* and the activity of G6Pase increased at the post-absorptive state (Mithieux et al., 2004b; Rajas et al., 1999). Since *gys1* mRNA abundance increased and glycogen accumulated at 3 HPF, the increased transcript levels of *g6pca1* and *g6pcb2* at 3 HPF might indicate an up-regulation of gluconeogenesis rather than glycogenolysis in the intestine of tilapia. The expression pattern of *g6pca1* and *g6pca2* in the intestine differed from those of liver, in which their mRNA abundance decreased during 1-3 HPF (Chen et al., 2017). Moreover, the mRNA level of *g6pca1* was comparable to that of *g6pca2* in the liver of tilapia (Chen et al., 2017). However, the mRNA abundance of *g6pca2* was superior to that of *g6pca1* in the intestine of tilapia.

Integrating the expression pattern of *pck* (*pck1* and *pck2*) and *g6pc* (*g6pca1* and *g6pca2*), it was suggested that IGNG was stimulated during 1-3 HPF at the state of nutrient assimilation. Thus, a simultaneous stimulation of intestinal glycolysis and gluconeogenesis was obtained during 1-3 HPF. To identify if the IGNG originated from some amino acids in the intestine of tilapia, we analyzed both the mRNA levels and enzyme activities of two important protein degradation indicators (Gpt and Got) in fish (Wade et al., 2014; Tian et al., 2015). As we expected, both the expression of cytosolic *got1* and mitochondrial *gpt2l* and *got2*, as well as their activities, increased during 1-3 HPF. In mammals, glutamine and alanine are precursors of glucose synthesized in the small intestine and liver, respectively (Rajas et al., 2000; Qian et al., 2015). In the liver of gilthead seabream *Sparus aurata*, an alternatively spliced transcript of cytosolic *gpt1* is associated with enhanced gluconeogenesis (Anemaet et al., 2008), and knockdown of *gpt1* improves hepatic carbohydrate metabolism (González et al., 2016). As far as we know, scarce information is available regarding the relevance of IGNG with the mRNA abundance or activity of aminotransferase in fish. In rats, portal sensing of IGNG is a mechanistic link in the diminution of food intake induced by a protein-enriched diet (Mithieux et al., 2005). The biological significance of postprandial stimulation of IGNG by some amino acids might be related to the regulation of food intake in tilapia, since one typical characteristic of fish diet is its richness in protein. If this is not the situation, amino acid-triggered IGNG would probably decrease the protein utilization efficiency and aggravate the postprandial hyperglycemia of fish. The biological significance of postprandial temporary stimulation of IGNG deserves further investigations in fish.

It was noteworthy to mention that intestinal uptake of glucose and glycolysis was induced during 1-3 HPF in tilapia, which was much earlier than that of carnivorous rainbow trout (about 6 h after the last meal) (Kirchner et al., 2008; Polakof et al., 2010). It seemed that the response time of the gut to utilize dietary carbohydrate was much faster in omnivorous tilapia, which at least partially explained the faster glucose clearance rates and higher carbohydrate utilization efficiency in omnivorous tilapia as compared with carnivorous fish.

## MATERIALS AND METHODS

### Experimental fish

One hundred male juvenile GIFT tilapia (100±9 g/fish) were obtained from Xiema Hatchery (Chongqing, China) and maintained in an indoor recirculation system consisted of two large rectangular glass tanks (700 liters). Fish were acclimatized to experimental condition for about one month, during which they were fed with a commercial extruded diet to apparent satiation manually two times daily at 9:00 h and 17:00 h. The analyzed proximate composition of the diet on the basis of dry matter was as follows: moisture, 6.86%; crude protein, 33.2%; crude lipid, 7.37%; ash, 9.49%; starch, 36.7%. Throughout the experimental period, water temperature, dissolved O_2_, pH and ammonia were maintained at about 28.1±1.8°C, 7.82±0.39 mg/l, 7.15±0.32 and 0.08±0.01 mg/l, respectively.

### Sampling procedure

After the acclimatization period, tilapia (173±20 g/fish) were food-deprived for 36 h, re-fed to satiation and subjected to sampling at 0, 1, 3, 8 and 24 HPF. At each time point, six fish were randomly selected and anaesthetized with 50 mg/l MS-222 (Sigma, St Louis, USA) for blood collection from caudal vein with heparinized sterile syringes. Then, anterior intestine samples of the six fish were dissected, immediately frozen in liquid nitrogen, and transferred to −80°C until used for real-time PCR analysis, enzymatic assay and glycogen determination. Blood samples were immediately centrifuged (4500 rpm, 10 min) at 4°C (Eppendorf centrifuge 5430 R, Hamburg, Germany), and plasma was separated and stored at −20°C until used for analysis of glucose level. Sampling time points of 0 (fasting for 36 h), 1, 3, 8 and 24 HPF represented five different values for fullness of gastrointestine during the period of digestion including almost an empty gut, a full stomach, a full stomach and foregut, an empty stomach and a full gut and trace food in gut, respectively (He et al., 2015). All experiments were conducted under the standard code of protocol for the Care and Use of Laboratory Animals in China. This research was approved by the Animal Ethics Committee of Southwest University.

### Proximate analysis of the diet

Proximate analysis consisted of determining moisture, protein, lipid and ash contents of the diet using standard methods (AOAC, 1995).Crude protein (*N*×6*.*25) was determined by the Kjeldahl method after an acid digestion using an auto-Kjeldahl System (Hanon, Jinan, China). Crude lipid was determined by the ether-extraction method. Moisture was determined by oven drying at 105°C for 24 h. Ash was determined using a muffle furnace at 550°C for 24 h. Starch content of the diet was determined by spectrophotometric determination of glucose after hydrolysis by heat-stable alpha-amylase and amylo-glucosidase (Sigma, St Louis, USA) (Hall, 2000).

### Plasma glucose determination

The level of plasma glucose was measured by spectrophotometric assay using a commercial kit (Nanjing Jiancheng Bioengineering Institute, Nanjing, China). The concentration of plasma glucose was expressed as mM/l.

### Glycogen and enzymatic activity assay

The level of glycogen and activities of hexokinase (Hk), phosphofructokinase (Pfk), phosphoenolpyruvate carboxykinase (Pck), glutamic-pyruvic transaminase (Gpt) and glutamic-oxaloacetic transaminase (Got) in the anterior intestine were determined by spectrophotometric assay using commercial kits (Nanjing Jiancheng Bioengineering Institute, Nanjing, China). The glycogen level is expressed as mg/g wet tissue. The determination of Hk activity is coupled with the reaction of glucose-6-phosphate dehydrogenase, and one unit (U) of its activity is defined as the generation of 1 mM NADPH per min at 37°C. One U of Pfk and Pck activity is defined the amount of enzyme that will generate 1 nM ADP and decompose 1 nM NADH per min at 25°C, respectively. One U of Gpt or Got activity is defined as the generation of 1 μM pyruvic acid per minute at 37°C. The protein concentration of the supernatant solution was determined by the biuret method, using bovine serum albumin as the standard.

### Identification of targeted genes

The complete cDNA sequences of representative genes involved with glucose transport, glucose metabolism and amino acid metabolism were successfully obtained from Ensembl (http://www.ensembl.org/) or NCBI (http://blast.ncbi.nlm.nih.gov/) databases. Sodium/glucose cotransporter (*sglt1*, also called as *slc5a1*) and facilitated glucose transporters (*glut1*, *glut2* and *glut4*, also called as *slc2a1*, *slc2a2* and *slc2a4*, respectively) were identified to evaluate glucose transport. For glucose metabolism, glycolytic hexokinase [*hk1*, *hk2* and *hk4*/glucokinase (*gck*)] and phosphofructokinase (*pfkl*: liver type of *pfk*; *pfkma*: muscle type a of *pfk*; *pfkmb*: muscle type b of *pfk*), glycogenic glycogen synthase (*gys1* and *gys2*) and gluconeogenic glucose-6-phosphatase catalytic subunit (*g6pca1*, *g6pca2*, *g6pc2* and *g6pc3*) and phosphoenolpyruvate carboxykinase (*pck1* and *pck2*) were targeted. Glutamic-pyruvic transaminase (Gpt) (*gpt1* and *gpt2l*: glutamic-pyruvic transaminase 2-like) and glutamic-oxaloacetic transaminase (*got1* and *got2*) were identified as indicators of amino acid metabolism. The Ensembl databases were searched to isolate specific gene sequences of other species such as zebrafish (*Danio rerio*), fugu (*Takifugu rubripes*), medaka (*Oryzias Latipes*), xenopus (*Xenopus tropicalis*), rat (*Rattus norvegicus*), human (*Homo sapiens*) etc., using the corresponding gene of tilapia as the query sequence. Phylogenetic tree of coding sequences of a targeted gene of the different species was constructed with the neighbor-joining method by MEGA 5, version 5.05 (Tamura et al., 2011) to confirm the accuracy of the sequence of tilapia genes isolated from Ensembl or NCBI database.

### RNA extraction, cDNA synthesis and qPCR procedure

Total RNAs from anterior intestine samples were extracted with RNAiso Plus reagent (TaKaRa, Japan), and the concentration and quality of the RNA were assessed by NanoDrop-2000. One μg of total RNA was used for DNase I (RNase-free) treatment and cDNA preparation using PrimeScript RT Master Mix Perfect Real Time Kit (TaKaRa, Japan). The primer sets used for real-time PCR analysis were designed using Primer Premier 6 (Premier Biosoft Int., USA) with at least one primer in each set flanking the intron-exon boundary to avoid amplification of the genomic DNA (Table S1). Before real-time PCR analysis, a regular PCR was carried out to test the specificity of the primer sets and preliminarily evaluate the relative expression of targeted genes using a pooled anterior intestine cDNA sample of five different sampling time points as the template. The 25 μl-reactions consisted of 12.5 μl Gotaq premix (Promega, Canada), 2.5 μl of diluted cDNA or PCR-grade water as negative control, 9 μl of PCR-grade water, and 0.5 μl of each 10 μM primer. The PCR reactions were initiated by denaturation at 95°C for 2 min, followed by 34 amplification cycles at 95°C for 30 s, 60°C for 30 s and 72°C for 30 s, and then 72°C for 10 min. The desired single target product was purified using the Agarose Gel DNA Fragment Recovery Kit Ver.2.0 (TaKaRa, Japan), and subcloned using the pGEM-T Easy Vector System (Promega, Canada). The plasmid was used for transformation of DH5α Competent Cells (Promega, Canada) and clones with inserts were sequenced (Invitrogen, Shanghai, China) to further confirm the identity of targeted genes.

The procedure of real-time PCR followed our previous study (Chen et al., 2017). The relative abundance of mRNA transcripts was evaluated using the formula: R=2^−ΔΔCt^ as described previously (Livak and Schmittgen, 2001). The geometric mean of the copy numbers of β-actin was used to normalize the gene expression data. The amplification efficiency of selected genes in this study varied from 96% to 103%.

### Statistical analysis

Results were presented as mean±s.d. of six replicates. Before statistical analysis, all data were tested for the normality of distribution (one-sample Kolmogorov–Smirnov test) and homogeneity of variances (Levene's test) among different treatments. When necessary, the data were root-transformed to meet the standard of normal distribution. Then, the data were subjected to one-way ANOVA and Tukey's multiple tests with minimal significant level at 0.05. All the statistical analyzes were done with SPSS 17 for Windows (SPSS Inc, Chicago, USA).
